# Median arcuate ligament syndrome with anomalous origin of the right inferior phrenic artery from the celiac artery: a case report

**DOI:** 10.3389/fsurg.2026.1744615

**Published:** 2026-02-27

**Authors:** Zhu Bin, Chen Jianfeng, Yang Zhipeng

**Affiliations:** Department of General Surgery, Suzhou BenQ Medical Center, Suzhou, Jiangsu, China

**Keywords:** anatomical variation, celiac artery, laparoscopic decompression, median arcuate ligament syndrome, right inferior phrenic artery

## Abstract

Median arcuate ligament syndrome (MALS) is a rare vascular disorder. We report the case of a 72-year-old man with MALS who was found to have an anomalous right inferior phrenic artery originating from the celiac artery (CA), which was not detected on preoperative computed tomography angiography. During laparoscopic decompression, this vessel was encountered unexpectedly. A temporary clamping test was performed, confirming no diaphragmatic or hepatic ischemia, after which the vessel was safely ligated. The CA was then fully decompressed. Postoperatively, the patient's symptoms resolved completely. This case underscores that significant vascular anomalies may only be revealed intraoperatively. The described clamping technique provides a simple and safe method for real-time functional assessment, aiding critical surgical decisions when managing unforeseen anatomical variations.

## Introduction

Median arcuate ligament syndrome (MALS) is a rare vascular disorder characterized by external compression of the celiac artery (CA) origin by the median arcuate ligament, potentially leading to chronic visceral ischemia. The classic triad of postprandial abdominal pain, weight loss, and an epigastric bruit is non-specific, often leading to initial misdiagnosis as more common gastrointestinal pathologies (1). Computed tomography angiography (CTA) and magnetic resonance angiography are the cornerstone imaging modalities for diagnosis in symptomatic patients (2). For those who require intervention, laparoscopic decompression of the CA with celiac ganglionectomy has become the standard therapeutic approach, demonstrating favorable clinical and hemodynamic outcomes (3, 4).

Precise preoperative vascular mapping is critical for surgical planning. The celiac artery typically exhibits a classic trifurcation, giving rise to the left gastric, common hepatic, and splenic arteries. However, significant anatomical variations exist, including patterns in which additional branches such as the inferior phrenic artery (IPA) directly originate from the CA (5, 6). While detailed imaging is imperative, some variants may still escape detection, potentially complicating intraoperative dissection and affecting procedural safety.

Despite meticulous preoperative imaging, subtle vascular anomalies may occasionally escape detection due to technical limitations or interpretative focus on the primary pathology. This article presents a retrospective case of MALS associated with an anomalous right inferior phrenic artery that was not identified on preoperative CTA but was encountered intraoperatively. We emphasize the critical role of dynamic surgical assessment—specifically through the techniques of temporary clamping and functional evaluation—in safely managing such unforeseen anatomical variations. This highlights a practical strategy to enhance procedural safety.

## Case description

**Figure 1:**
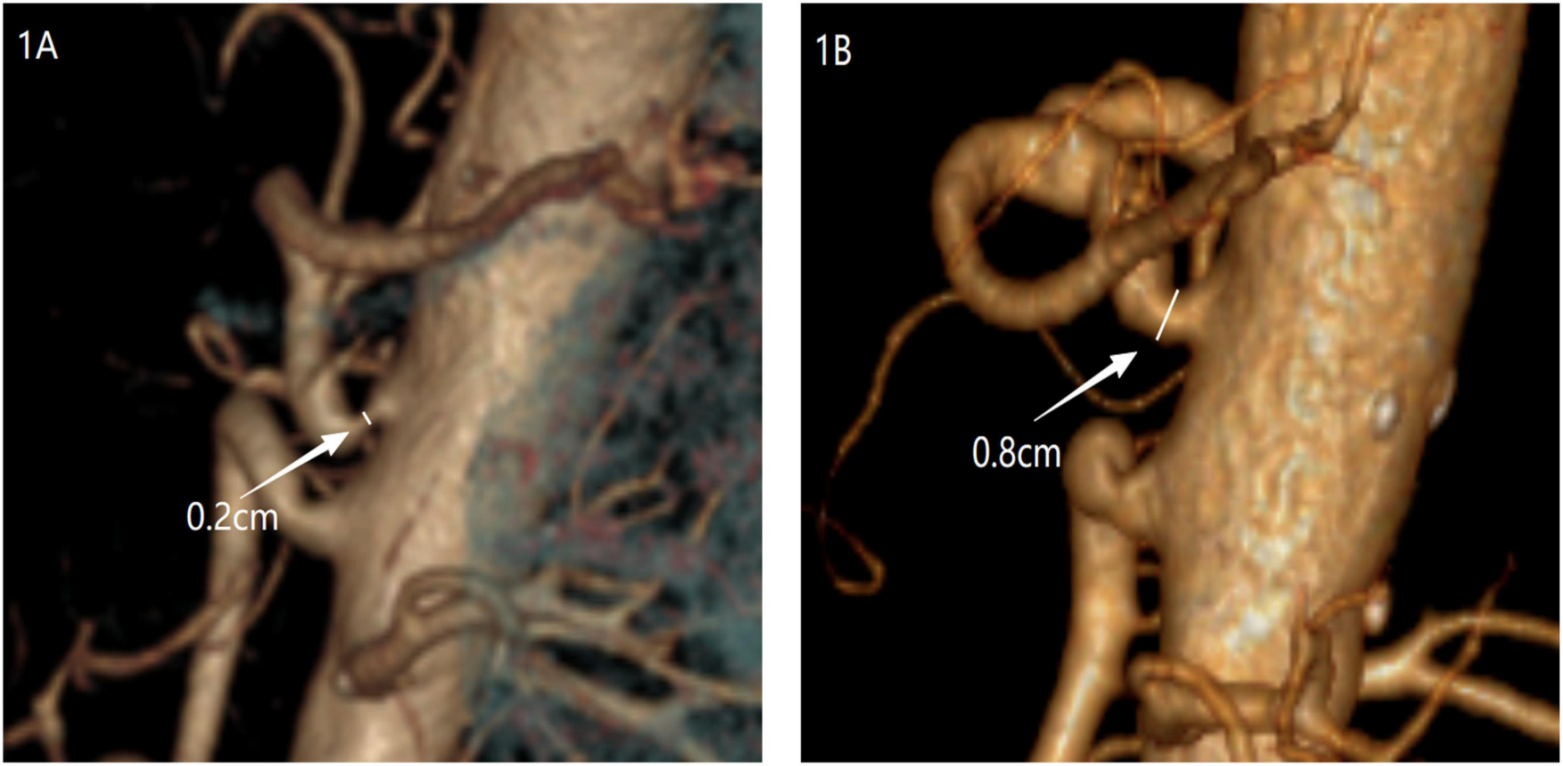
**(A)** CTA reveals a V-shaped narrowing at the origin of the CA with distal dilation, with the narrowest diameter measuring approximately 0.2 cm. **(B)** CTA shows that the origin of the CA is markedly dilated compared with prior imaging, with the narrowest segment measuring approximately 0.8 cm.

**Figure 2:**
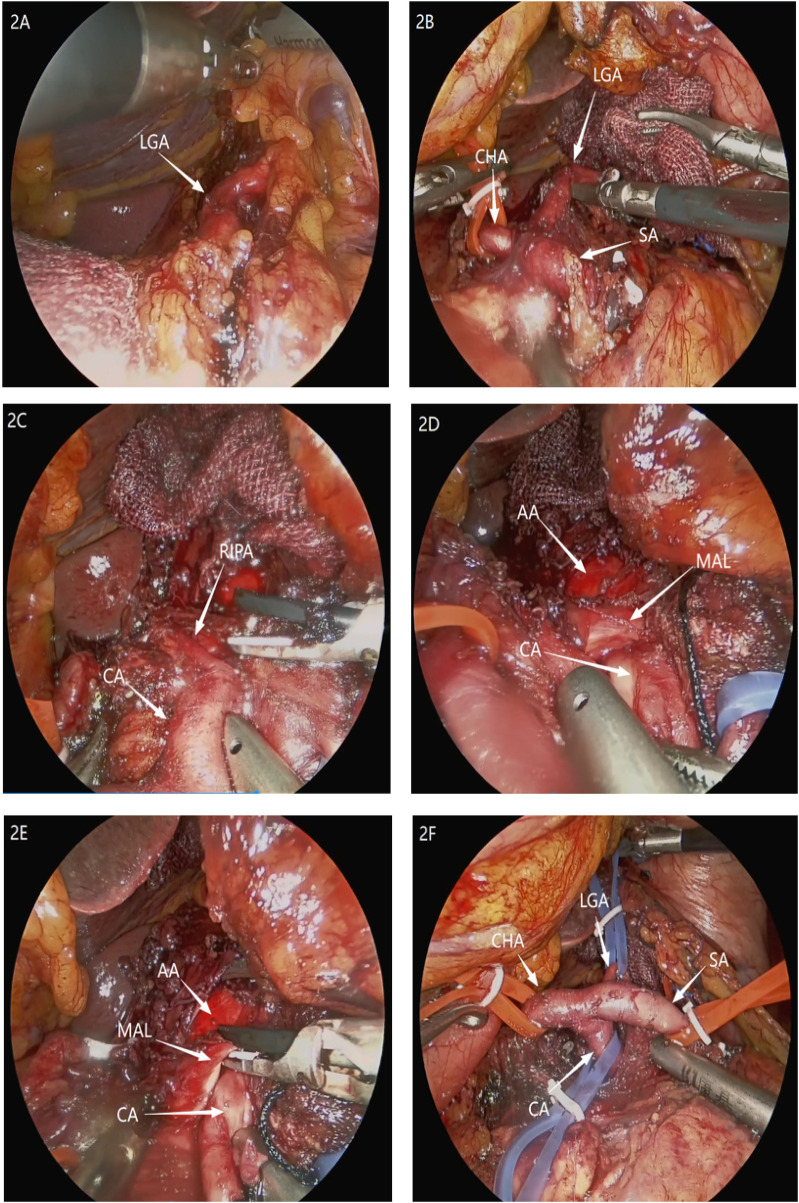
**(A)** Suspension of the stomach to expose the LGA at the superior border of the pancreas. **(B)** The SA and CHA are dissected proximally. **(C)** The anterior wall of the CA is dissected, revealing a variant right inferior phrenic artery originating from its origin. **(D)** Exposure of the CA root to the AA reveals compression of its origin by the MAL. **(E)** The MAL compressing the CA is transected using a Harmonic scalpel. **(F)** The skeletonized CA and its branches.

**Figure 3:**
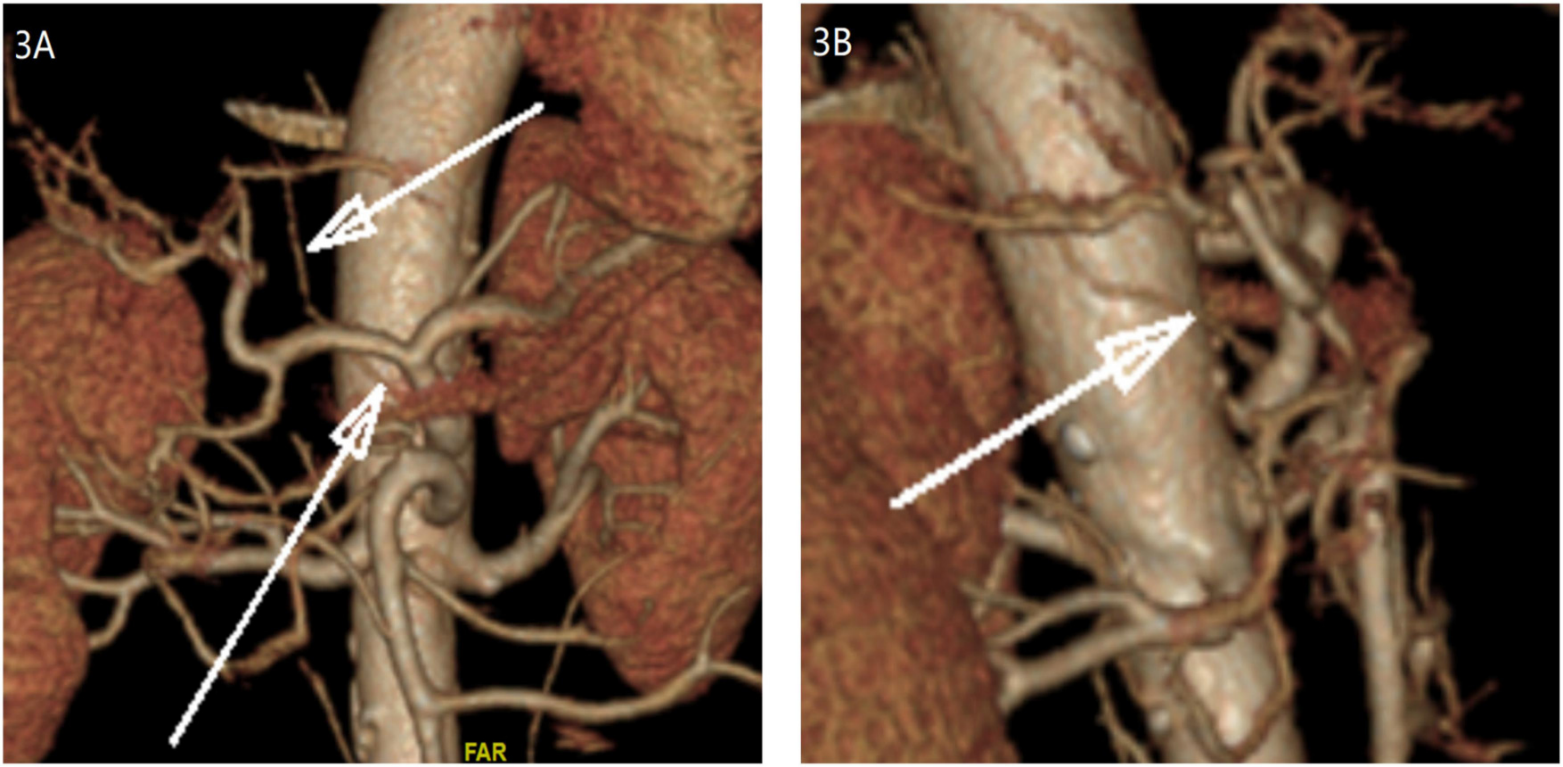
Retrospective CTA confirmation of the variant right inferior phrenic artery. **(A)** Coronal view showing the anomalous RIPA (white arrows) originating from the celiac artery and coursing toward the right diaphragm. **(B)** Sagittal view with a single arrow indicating the proximal segment of the variant vessel.

A 72-year-old man was admitted with a 2-year history of recurrent upper abdominal pain and approximately 10 kg of weight loss. Physical examination of the abdomen was unremarkable. Initial investigations, including laboratory tests and gastroscopy, revealed only esophagitis and gastritis. As his symptoms persisted despite acid-suppressive therapy, these findings were deemed insufficient to explain the clinical presentation. To evaluate for a neurovascular etiology, computed tomography angiography (CTA) was performed. It demonstrated significant stenosis at the origin of the CA, consistent with a diagnosis of MALS (Figure 1A). The patient subsequently underwent laparoscopic median arcuate ligament (MAL) decompression and celiac ganglionectomy. During dissection of the CA root, a small-caliber vessel was encountered that obstructed surgical exposure. This vessel originated directly anterior to the CA and coursed superiorly and posteriorly toward the right diaphragm, suggestive of an anatomical variant of the right inferior phrenic artery (RIPA). The vessel was temporarily clamped at its origin for 5 min. During this period, diaphragmatic color and contractility, hepatic surface perfusion, and systemic hemodynamics were closely monitored; no changes were observed in diaphragmatic color or contractility, with no hepatic perfusion deficit. The vessel was consequently ligated and divided. Following this, the compressing MAL fibers were completely incised, and the surrounding neural tissue was dissected to fully expose the aorta deep to the CA (Figure 2). Within this exposed area, no arterial branch corresponding to a left inferior phrenic artery (LIPA) arising from the CA was identified. Postoperatively, the patient reported significant relief of abdominal pain by the 10th day. Follow-up CTA showed marked improvement in the patency of the CA (Figure 1B). In light of this intraoperative discovery, consultation with the radiology department was undertaken. A targeted multiplanar reconstruction of the preoperative CTA was performed, with focused analysis on the vessels around the CA. This subsequent review clearly delineated the anomalous RIPA encountered during surgery (Figure 3). At the 6-month follow-up, the patient reported complete resolution of abdominal pain.

## Discussion

MALS is a relatively rare condition, with an estimated incidence of 1.74%–4.0%. It is more common in lean women aged 40–60 years, with a male-to-female ratio of about 1:4 (7, 8). The classic manifestations include postprandial abdominal pain and weight loss, which may be persistent or episodic. Some patients experience relief in the prone position or with deep inspiration (9, 10).

The pathogenesis of MALS is classically attributed to extrinsic compression of the CA by the MAL. Historically, this condition has been explained by a high origin of the CA or a low diaphragmatic insertion, often influenced by congenital factors (4). In essence, it is considered an anatomic compression syndrome resulting from the aberrant spatial relationship among multiple local structures, including the MAL, CA, celiac nerve plexus, and diaphragmatic crura (11). During early embryonic development, the CA and AA are formed by paired segmental arteries that converge and undergo caudal migration. It has been hypothesized that aberrant migration may lead to a high-lying CA, predisposing it to compression (11). However, the primacy of this embryological-anatomical hypothesis has been questioned. Recent imaging and anatomical studies have not offered supportive evidence for a superiorly positioned CA as the sole causative factor. Instead, other morphological parameters, such as the CA angle, may play a more critical role (12, 13).

These debates underscore the inherent complexity and individual variability of the peri-celiac anatomy, which extends beyond the ligament–artery relationship to include variant vascular branches. Indeed, due to such anatomical variations, a configuration predisposing to compression is present in approximately 10%–24% of the population. However, about 85% of these individuals remain asymptomatic, which is largely attributable to robust collateral circulation between the CA and the superior mesenteric artery (SMA), which compensates for potential vascular insufficiency (7, 14, 15). The development of symptomatic MALS, therefore, likely requires additional elements beyond mere anatomic predisposition, such as significant neurogenic stimulation of the celiac plexus (12). Furthermore, the correlation between the degree of stenosis, hemodynamic improvement after surgery, and clinical symptoms is complex and not always parallel, supporting a multifactorial pathophysiology (16).

The CA, as the principal arterial supply to the foregut, most commonly exhibits a classic trifurcation into the left gastric, common hepatic, and splenic arteries. However, variations in its branching pattern are well documented and can include additional branches such as the IPA originating directly from the CA, forming quadrifurcation or other complex patterns (5). These anatomical variations have a clear embryological basis. The abdominal aorta (AA) develops through the fusion of the paired dorsal aortae, while their ventral splanchnic branches coalesce into a midline vascular plexus. A process of selective regression then consolidates this plexus into the three definitive ventral arteries: the CA, SMA, and inferior mesenteric artery (17). In parallel, the IPA and other lateral splanchnic arteries develop from the aortic wall. Variations in the persistence, regression, or fusion of these embryonic vascular channels account for the diversity observed in adult anatomy (18), as exemplified by the present case. An alternative embryological hypothesis suggests that anomalous origins of the IPA may arise from the ventral segments of the primitive aorta, potentially at levels shared with the developing CA (19). In our patient, the anomalous RIPA originating from the CA likely resulted from abnormal persistence of a communication between the developing CA and an early adrenal-phrenic vascular channel.

The IPAs are a pair of vessels that typically originate from the AA or the CA, either independently or via a common trunk. (20). However, this anatomical structure exhibits significant variability. A meta-analysis by Whitley et al. (*n* = 4,208) indicated that the RIPA most commonly originates from the abdominal aorta, followed by the celiac artery, whereas the LIPA originates from either vessel with nearly equal probability (21). Ekingen et al.'s study of 1,000 patients further confirmed this high variability, even documenting cases with only a single superior mesenteric artery or the complete absence of such arteries (22). From what is known thus far, there is great anatomic variability in the origin of the CA and IPA. As seen in our patient, an accessory RIPA arising from the CA was not clearly delineated on preoperative imaging and was discovered incidentally during surgical exploration. Upon dissecting the CA origin, we identified an arterial branch coursing superiorly and posteriorly toward the right diaphragmatic crus. To achieve complete decompression of the CA, this artery required either preservation or division. Given that the identified vessel was only suspected to be the RIPA and its supply territory remained uncertain, its hemodynamic significance needed assessment before deciding whether to preserve or transect it. Evidence indicates that although the intestinal mucosa is metabolically active and susceptible to ischemia, rapid repair mechanisms can be activated following transient ischemia (23). Skeletal muscle, including the diaphragm, demonstrates greater ischemic tolerance than the highly metabolic intestinal mucosa. Furthermore, in hepatobiliary surgery, the Pringle maneuver for inflow control has a well-established safe duration of 15–20 min or longer (24). Therefore, we adopted a more conservative strategy of temporary clamping for 5 min. No changes in diaphragmatic contractility or hepatic coloration were observed, indicating sufficient collateral perfusion. Given its non-critical function and to optimize surgical access, the vessel was ligated and divided. Subsequently, we successfully completed laparoscopic decompression of the CA with excision of the celiac ganglion. Given the absence of a left-sided variant within our surgical field, we conclude that the LIPA most likely originated conventionally from the AA in this patient.

In this case, a variant RIPA originating from the CA was discovered intraoperatively and later confirmed on retrospective imaging review. Preoperative CTA failed to detect this vessel, primarily due to its small diameter and overlapping branches at the origin, which complicated identification. Furthermore, the interpreter's experienced-based focus was directed toward the stenosis of the main celiac artery, and insufficient awareness of common anatomical variants in this region also contributed to the oversight. Previous research by White et al. has indicated that CTA carries a certain probability of false negatives when imaging small-caliber vessels (25). The present case further corroborates that viewpoint from a clinical perspective.

## Conclusion

In conclusion, we report a rare case of MALS associated with an anomalous RIPA originating from the compressed CA, which was not identified on preoperative CTA but was discovered intraoperatively. This finding highlights two critical points for surgical practice: First, even high-resolution preoperative imaging may fail to reveal clinically relevant vascular variations. Second, when such anomalies are encountered, a simple intraoperative temporary occlusion test provides a safe and reliable method to assess their hemodynamic significance in real time, guiding definitive management. This case underscores the importance of surgical vigilance and dynamic assessment in achieving both safety and completeness of decompression in MALS surgery.

## Data Availability

The original contributions presented in the study are included in the article/Supplementary Material, further inquiries can be directed to the corresponding author.
